# Private Healthcare Expenditure in China: A Regional Comparative Analysis

**DOI:** 10.3390/healthcare9101374

**Published:** 2021-10-14

**Authors:** Shangguang Yang, Danyang Wang, Lu Xu, Chunlan Wang, Xi Yang, Kevin Lo

**Affiliations:** 1 Economic Development Institute, East China University of Science and Technology, Shanghai 200237, China; sgyang@ecust.edu.cn (S.Y.); aliceluuu@163.com (L.X.); 2 School of Business, East China University of Science and Technology, Shanghai 200237, China; dywang007@163.com; 3 Chinese Modern City Research Center, School of Social Development, East China Normal University, Shanghai 200062, China; clwang@soci.ecnu.edu.cn; 4 David C. Lam Institute for East-West Studies, Hong Kong Baptist University, Hong Kong 999077, China; xiyang@hkbu.edu.hk; 5 Department of Geography, Hong Kong Baptist University, Hong Kong 999077, China

**Keywords:** private healthcare expenditures, regional differences, influencing factors, China

## Abstract

Private (out-of-pocket) healthcare expenditure (PHCE) is a complex phenomenon that is shaped by many different factors. In this paper, we analyzed the influencing factors of PHCE in China, with a specific focus on regional differences. We found that old-age dependency ratio, income, and education have significant impacts on PHCE in all regions, whereas public HCE, number of beds in medical institutions, and economic development levels have significant impacts only in some regions. The results indicate that the government should pay attention to regional inequality and implement targeted adjustments for improving the health service system. In particular, we recommend: (1) monitoring regional inequality in PHCE and other healthcare issues to unmask geographical differences in healthcare interventions; (2) adopting regional-specific policy measures—the government should divert some resources from eastern to western and central regions to increasing the support for public health undertakings and improve the quality of the local health services while providing matching medical resources by targeting the needs of the residents; (3) paying more attention to the healthcare demand of the elderly population; and (4) improving the education level of residents to improve public health and avoid high PHCE.

## 1. Introduction

Understanding private healthcare expenditure (PHCE) is essential for policymakers in order to identify problems associated with the accessibility and affordability of healthcare [1]. These problems, if not handled properly, may become a threat to economic development, social cohesion, and political stability [2]. In China, PHCE is rapidly rising—PHCE in 2018 increased by 23% compared with 2013, reaching 1685 yuan per capita and accounting for 8.49% of household consumption expenditure [3]. However, there is limited empirical analysis over this important trend, especially given that China is such a vast country with significant regional differences in socioeconomic development. While China has seen rapid economic growth over the past two decades, such development has also contributed to regional inequality—the eastern coastal region has the highest level of economic development, followed by the central and western regions [4,5]. Consequently, while overall medical resources have gradually improved [6], regional differences in healthcare can be detected in the spatial distribution of healthcare resources [7]. For instance, residents living in eastern China use more outpatient and in-patient services than those living in central and western China [8]. Accessibility to medical facilities is better in coastal regions than in western and inland regions [9]. In addition, self-rated health is better for individuals from the eastern region, and the regional disparities increase with age, suggesting that an aging society may reinforce the phenomenon of regional inequality [10]. The divide between rural and urban areas is also substantial [11], and PHCE has become an important source of transient poverty in rural China [12]. Worryingly, the inequalities of healthcare resources is worsening in recent decades [13].

These studies suggest that PHCE in China could be affected by regional differences in socioeconomic factors, demographic factors, and the level of government support. To support evidence-based policymaking, this study offers a regional comparative analysis on the influencing factors of PHCE in eastern, central, and western China. Our study supplements the existing studies conducted at the national level. The principal research question is: What are the influencing factors of PHCE in China at the regional levels? To clarify the terminologies before proceeding any further, HCE in this study is classified into two types: private and public [14]. Private HCE refers to out-of-pocket payments by individuals [15], whereas Public HCE refers to the investment made by the government, including public health spending, government subsidies to disadvantaged social groups, the government’s contribution to medical schemes, and medical insurance for civil servants and employees of public institutions [16].

The rest of this paper is organized as follows. Section 2 reviews the literature on the influencing factors and models of PHCE. The methodology and materials are outlined in Section 3. The results are presented and discussed in Section 4 and Section 5. Finally, concluding remarks are presented in Section 6, which also highlights the policy implications of the findings.

## 2. Influencing Factors and Models of PHCE

At the household and individual level, a diverse set of factors are found to be closely linked to PHCE: household socioeconomic factors, such as income level and its distribution, level of education, and employment status [17,18], demographic factors, especially age and gender [19,20], households’ location—whether it is located in a rural or urban area [21], and health status, which is measured by factors such as the number of inpatients in the household, number of members with chronic diseases, and adoption of healthy lifestyles [22,23,24,25]. Studies on the macro-level factors of PHCE focus on broader socioeconomic and political factors. Usman, et al. [26] found that the macroeconomic factors such as growth in the gross domestic product (GDP) and foreign direct investment (FDI) consistently showed a positive impact on PHCE. Karyani et al. [27] discovered that freedom of the press and democracy would increase PHCE. The link between environmental quality and PHCE has been highlighted by Toplicianu [28], who argued that environmental deterioration increases the incidence of disease and, therefore, PHCE. Reich et al. [29] have revealed that PHCE is significantly related to the densities of specialist physicians and dispensing doctors, the proportion of managed medical care, and medical and technological progress. However, Sorenson et al. [30] argued that the relationship between medical service availability and expenditure is complex, sometimes conflicting, and often depends on different factors, such as the availability of other interventions and the number of patients. Furthermore, the impact of technology on costs differs across technologies. For example, some technologies (e.g., cancer drugs, invasive medical devices) have significant financial implications, while others are cost-neutral or cost-saving. A multilevel analysis of 28 countries has shown that the linkage between public HCE and inequalities in access to health care is complex, and public HCE at a national level does not moderate the relationship between income and PHCE [31].

Studies that integrated macro- and micro-level factors typically found that both are important, but to a different degree. Thornton and Beilfuss [32] found that the most important factors that affect PHCE are provider availability, income, excessive drinking, insurance coverage, and the proportion of the elderly and African-American populations. de Meijer, et al. [33] have demonstrated that the availability of medical technology is the most important driving force of PHCE, and it has a strong interaction with aging and health condition.

The Grossman’s model is an influential statistical tool for the modeling of PHCE [34]. Based on the assumption that individuals simultaneously optimize health and wealth [35,36,37], Grossman constructed a theoretical model for analyzing the healthcare demand, which has also been labeled as the human capital model of the demand for health [38]. Grossman tailored the model to suit the healthcare context and stressed that health production requires both goods and time [39]. Over the years, a series of debates have been focused on the Grossman model, including criticisms and supports [40,41,42,43]. Several attempts have been made to optimize the model. Muurinen [44] developed a generalized version of Grossman’s model by using a different conceptual framework. Jones et al. [45] developed an integrated model synthesizing the Grossman and the Becker-Murphy models of health and addiction [46]. These efforts indicate that there is no universal model applicable to any circumstance. Meanwhile, Grossman’s health demand model has been empirically applied and tested by various studies. For instance, Fukui and Iwamoto [47] have studied the PHCE and the health outcome of the Japanese population. Abdul-Rahman [48] studied the demand for physical activity of the American elderly population. Gu et al. [49] demonstrated the correlation between human health damages and air pollution in China. Hartwig and Sturm [50] tested the model with macroeconomic panel data from 29 OECD countries.

## 3. Materials and Methods

### 3.1. Data and Selection of Variables

Taking into account the existing research on the factors affecting PHCE, this study developed a PHCE model based on the Grossman’ health demand model. The variables are shown in Table 1. The total dependency ratio can be divided into two: old-age dependency ratio (ODR, over 65) and youth dependency ratio (YDR, under 15). ODR offers information concerning the welfare system, and YDR represents the relation between the potentially active person and those who depend directly on family income [51,52]. To reduce the impact of heteroscedasticity, the logarithm of all variables was taken to make the data more stable. The model is listed as follows:

(1) $$lnPHCE=\beta_{0}+\beta_{1}lngdp+\beta_{2}lnodr+\beta_{3}lnydr+\beta_{4}lnincome+\beta_{5}lnedu+\beta_{6}lngov+\beta_{7}lnP+\beta_{8}lnbed+\mu$$

This study uses panel data from 31 provinces, municipalities, and autonomous regions in China from 1998 to 2017. The data are classified into three different regions: eastern, central, and western. Data were mainly drawn from China Health Statistical Yearbook, China Statistical Yearbook, and China Population and Employment Statistical Yearbook [3,53,54].

### 3.2. Regression Model Selection

To obtain the most accurate results, we choose the optimal model through the following empirical testing. First, comparing the mixed-regression and the fixed-effects model, in the fixed-effect F test, the P values are all 0.0000, indicating the superiority of the fixed-effects model over the mixed-regression model. We use the Hausman test [55] to compare the fixed-effects and the random-effects model. The hypothesis of the Hausman test is $H_{0}:u_{i}\;is\;not\;related\;to\;x_{it},\;z_{i}$, which means, in other words, that the random-effects model is an optimal model. If $H_{0}$ is not established, the fixed effects are more effective.

Table 2 shows that the null hypothesis is rejected at the national level as well as the eastern, central, and western regions. Therefore, we select the fixed-effects model for regression analysis. The results of the Hausman test revealed that the null hypothesis is rejected at the national level; thus, we select the fixed-effects model as the best model for regression analysis. We also choose this model for regional analysis because the eastern, central, and western regions are all significant at the 1% level, which also rejected the null hypothesis.

## 4. Results

### 4.1. Descriptive Statistical Results

As shown in Table 3, the eastern region has the highest level of PHCE, followed by the central region and the western region. The average value of GDP in the eastern region is significantly greater than that in the central region and much larger than that of the western region; meanwhile, the internal economic development of the eastern region has the largest difference. The average education level of residents in the eastern region is the highest. In terms of residents’ disposable income, the average value in the eastern region is by far the highest—almost double that of the central and western regions. The eastern region has the highest public HCE, followed by the central region and the western region. The average public HCE in the eastern region is about three times the national level, indicating that the eastern government has more income and pays more attention to medical and health undertakings. The western region has the lowest number of beds in medical institutions, indicating that the medical and health resources in the western region are relatively scarce. Turning to demographic factors, ODR is the highest in the eastern region and lowest in the western region, whereas YDR is the highest in the western region and lowest in the eastern region. This shows that eastern China has the oldest population while western China has the youngest.

### 4.2. Regression on Influencing Factors

#### 4.2.1. National Level

The results of the fixed-effects model using national-level data reveal that ODR, residents’ disposable income, residents’ education level, number of beds in medical institutions, and overall economic development level are significant factors, while the YDR, public HCE, and medical consumer price index are not significant (Table 4).

The regression results indicate that PHCE is mainly affected by ODR, disposable income, education level, number of beds in medical institutions, and overall economic development level. The regression Equation (2) is shown below:

(2) lnPHCE = −1.26 + 0.5lnodr + 0.53lnincome + 0.1lnedu − 0.24lnbed + 0.36lngdp + μ

Meanwhile, the number of beds in medical institutions has a significant negative impact on PHCE. An increase in the number of beds suggests that more medical services are being provided, which provides residents with more choices. This development allows residents to choose more cost-effective health care services, which may reduce PHCE.

#### 4.2.2. Eastern China

The main factors that affect PHCE in the eastern region are ODR, disposable income of the residents, level of education, public HCE, and number of beds in medical institutions. The obtained regression Equation (3) is as follow:

(3) lnPHCE = −1.34 + 0.33lnodr + 0.92lnincome + 0.27lnedu − 0.08lngov − 0.34lnbed + μ

Unlike the results from the national-level analysis, GDP is not a significant factor, suggesting that the economic situation in the region has always been good during the studied period; thus, it has little effect on PHCE. Also, unlike the national situation, PHCE is significantly affected by public HCE. With the increase in government expenditure in healthcare, PHCE has decreased significantly, indicating that public investment in medical and health services has reduced the burden of PHCE in this region.

The impact of ODR in the eastern region is smaller than at the national level. The reason for this low impact may be the better medical security system in the region: the HCE of the elderly can be better covered by the medical security system.

#### 4.2.3. Central China

For the central region, the main factors affecting PHCE are ODR, residents’ disposable income, level of education, and number of beds in medical institutions. The following regression Equation (4) is obtained:

(4) lnPHCE = −4.94 + 0.29lnodr +1.24lnincome + 0.09lnedu − 0.48lnbed + μ

The level of economic development is not a significant factor, suggesting that the economic development level of the central region has been relatively stable. The impact of the ODR and the education level of the residents on PHCE is lower than that in the whole country. Furthermore, the impact of income and the number of beds in medical institutions are much higher.

#### 4.2.4. Western China

For the western region, the main factors affecting PHCE are ODR, residents’ disposable income, level of education, and level of regional economic development. The following regression Equation (5) is obtained:

(5) lnPHCE= −1.03 + 0.56lnodr + 0.25lnincome + 0.13lnedu + 0.48lnbed + μ

The number of beds in medical institutions is a significant factor at the national level but not in the western region, suggesting that the western region has fewer healthcare resources, and the quality is relatively low. When the residents have serious healthcare demands, they tend to go to central or eastern regions, where offer richer and higher-quality medical services. ODR, residents’ education level, and level of GDP have a greater impact on PHCE in the western region than the national level, while the impact on residents’ income is lower.

## 5. Discussion

Healthcare inequality between different population groups and regions is not unique to China but is a global issue [56]. However, different dynamics contribute to such inequality in different countries. In India, for instance, religion and caste have a strong influence on society’s enormous healthcare disparities [57]. In Bangladesh, regional inequality in healthcare is closely related to wealth-related disparities, such as the socioeconomic inequality in the utilization of healthcare services [58]. A study conducted in Italy and Spain found that regional inequalities in health system satisfaction and PHCE can be attributed to the different ways the healthcare system is designed and managed [59]. For China, the most fundamental aspects underpinning regional inequality are that of rural–urban and inland–coastal inequalities, which are themselves the product of various government policies [60,61]. As such, China offers a unique case study to understand the complex issues of PHCE and inequality.

The findings from this study show that factors of PHCE are not equally influential across different regional contexts. On the one hand, there are a number of factors that are shared by the three regions. First, there is a significant positive correlation between ODR and PHCE in all three regions, although the degree of impact differs. Second, residents’ income is the greatest factor in all regions except the western region, suggesting that the income level of residents in the western region is consistently low and medical facilities highly insufficient. Third, residents’ education level is significantly related to PHCE, the importance of education is relatively high in the eastern region but is relatively low in the central and western regions, again reflecting the lower level of education in these regions. 

On the other hand, there are factors that have different impacts on different regions. There are significant regional differences in the impact of public HCE and number of beds in medical institutions on PHCE. The number of beds in medical institutions showed a significant negative effect in the eastern and central regions but did not show a significant impact in the western region. This is again mainly because of the lack of medical facilities in the western region. Public HCE shows a significant negative impact in the eastern region, indicating that current public HCE has a substitution effect on PHCE. However, in the central and western regions, public HCE has not been shown to exert a significant impact, suggesting a lack of public investment in health care in these areas. Overall, these findings show that the regional differences in PHCE in China are associated with regional inequality in socioeconomic development and the quality and availability of healthcare services.

## 6. Conclusions

This study examined factors that shape PHCE in China from a regional perspective. The findings suggest that it is critical to address the regional differences and implement targeted adjustments for improving the medical service system. We offer five detailed policy recommendations. First, monitoring regional inequality in PHCE and other healthcare issues is a helpful practice to unmask geographical differences in healthcare interventions and offer important evidence for healthcare policies [62]. For example, the public health development index, a composite index including public health infrastructure, services, behavioral risk factors, and health outcomes, has been introduced in Indonesia to quantify subnational regional inequality [63]. Second, given the different income levels in various regions, regional-specific policy measures should be adopted. Our results point to the allocation of medical resources and healthcare investment should be different in different regions. In the central and western regions, the government should focus on increasing the support for public health undertakings and improve the quality of the local health services, while providing matching medical resources by targeting the needs of the residents. Given the generally higher income level of the eastern region, the government should divert some resources to western and central regions, prevent residents from misusing medical resources, and formulate guiding policies to help residents choose medical services rationally. Third, as the population is aging rapidly in China, more attention is needed to the healthcare demand of the elderly population. It is necessary to optimize the policies for the elderly in the current medical system. The government’s investment in basic medical insurance for the elderly should be established in multi-level and multi-dimensional dimensions. Fourth, improving the education level of residents is important. Better education not only facilitates good health habits, such as regular medical check-ups, which can help control disease and avoid high PHCE.

## Figures and Tables

**Table 1:** Assignment of variables.

Variable Type	Variable	Description
Dependent	PHCE	Private healthcare expenditures
Independent	GDP	Gross domestic product
	ODR	Old-age dependency ratio
	YDR	Youth dependency ratio
	Income	Resident income, refer to the actual per capita disposable income of residents
	Edu	Educational level, refer to the proportion of the population of college and above in the total population
	Gov	Government’s investment in health services
	Price	Healthcare prices, refer to medical consumer price index
	Bed	Number of beds in medical institutions

**Table 2:** Hausman test results.

Region	Chi-Square Statistics	Conclusion
National	56.09	Significant at the 1% level
Eastern	37.61	Significant at the 1% level
Central	92.85	Significant at the 1% level
Western	33.99	Significant at the 1% level

**Table 3:** Descriptive results.

Variable	Region	Mean	Standard Deviation	Maximum	Minimum
PHCE	National	803.31	516.37	2900.00	91.92
	Eastern	922.56	555.02	2900.00	133.86
	Central	755.66	494.47	2165.46	91.92
	Western	726.04	474.29	2140.81	136.90
GDP	National	11,969.33	13,956.03	89,705.20	91.50
	Eastern	19,098.82	18,678.47	89,705.23	442.13
	Central	11,227.04	9303.18	44,552.83	1577.05
	Western	5928.82	6586.46	36,980.22	91.50
ODR	National	12.15	2.78	21.90	6.13
	Eastern	13.08	2.52	21.90	8.26
	Central	12.05	2.34	19.10	6.93
	Western	11.38	3.04	20.60	6.13
YDR	National	25.84	8.34	57.78	9.60
	Eastern	21.45	7.66	46.68	9.60
	Central	25.84	7.23	41.60	11.89
	Western	29.87	7.58	57.78	13.74
Income	National	16,311.85	10,340.44	62,595.74	4010.00
	Eastern	20,212.68	12,423.44	62,595.74	4617.20
	Central	14,171.95	8346.02	33,947.94	4098.70
	Western	14,162.68	8212.99	35,670.00	4010.00
Edu	National	0.09	0.07	0.69	0.01
	Eastern	0.12	0.09	0.48	0.02
	Central	0.07	0.06	0.69	0.01
	Western	0.07	0.04	0.18	0.01
Gov	National	146.61	185.26	1307.56	1.49
	Eastern	176.90	215.25	1307.56	1.49
	Central	158.25	183.38	836.66	8.39
	Western	111.19	147.91	831.46	1.92
Price	National	101.79	3.55	137.1	90.30
	Eastern	101.56	3.31	115.8	90.30
	Central	101.67	3.19	110.9	92.10
	Western	102.09	3.97	137.1	92.30
Bed	National	14.85	10.83	58.48	0.61
	Eastern	17.13	12.02	58.48	1.78
	Central	18.13	9.48	55.90	8.40
	Western	10.57	9.03	56.35	0.61

**Table 4:** Regression results of fixed effect model in China.

Variable	National			Eastern			Central			Western		
	c	sd	t	c	sd	t	c	sd	t	c	sd	t
lnodr	0.500 ***	0.055	9.14	0.333 ***	0.082	4.05	0.285 **	0.116	2.45	0.560 ***	0.105	5.33
lnydr	0.004	0.049	0.09	−0.007	0.093	−0.07	0.121	0.090	1.35	0.051	0.068	0.76
lnincome	0.531 ***	0.052	10.12	0.923 ***	0.146	6.32	1.235 ***	0.167	7.38	0.253 ***	0.059	4.26
lnedu	0.098 ***	0.024	4.02	0.275 ***	0.059	4.66	0.091 **	0.039	2.35	0.138 ***	0.036	3.84
lngov	−0.025	0.025	−1.01	−0.086 ***	0.032	−2.67	−0.052	0.068	−0.76	−0.065	0.053	−1.22
lnp	−0.258	0.201	−1.28	−0.151	0.338	−0.45	−0.553	0.379	−1.46	−0.003	0.003	−1.16
lnbed	−0.236 ***	0.058	−4.09	−0.342 ***	0.100	−3.44	−0.476 ***	0.111	−4.30	−0.020	0.086	−0.23
lngdp	0.356 ***	0.051	7.05	0.084	0.106	0.79	0.063	0.141	0.44	0.482 ***	0.085	5.70
C	−1.257	0.995	−1.26	−1.336	1.674	−0.80	−4.942	1.066	−4.64	−1.034	0.648	−1.60
$R^{2}$	0.9403	*p*	0.00	0.9431	*p*	0.00	0.9687	*p*	0.00	0.9445	*p*	0.00

Note: c is coefficient; sd is standard deviation; t is t-value; C is constant; *p* is *p*-value of F-test; ***, ** and * indicate that the *p* value is significant at the levels of 1%, 5% and 10% respectively.
